# The effect of the I1307K *APC* polymorphism on the clinicopathological features and natural history of breast cancer

**DOI:** 10.1038/sj.bjc.6690775

**Published:** 1999-11

**Authors:** Z Q Yuan, L R Bégin, N Wong, J-S Brunet, M Trifiro, P H Gordon, L Pinsky, W D Foulkes

**Affiliations:** Departments of Medicine, Surgery, Oncology, Pathology andCancer Prevention Research Unit, Sir MB Davis-Jewish General Hospital, 3755 Cote Ste Catherine, Montreal, Quebec, H3T 1E2, Canada; Centre for Research in Women’s Health, University of Toronto, Ontario, Canada; Departments of Medicine and Human Genetics, McGill University, Montreal, Quebec, Canada

**Keywords:** breast cancer, APC, I1307K, polymorphism, survival

## Abstract

The I1307K polymorphism in *APC* has been found to predispose to colorectal cancer in Ashkenazi Jews, and has recently been associated with an increased risk for breast cancer in the same population. In that study, we genotyped 205 paraffin-embedded breast cancers from Ashkenazi Jewish women diagnosed below the age of 65. We now present an extended analysis, with clinicopathological correlations between carriers of I1307K and non-carriers. Twenty-four of 209 cases (11.5%, 95% confidence interval 7.5–16.6) were found to carry the I1307K polymorphism. When stratifying the data by other relevant clinicopathological variables, we observed no association between the presence of this polymorphism and age at diagnosis (*P* = 0.52), grade (*P* = 0.074), tumour size (*P* = 0.99), lymph node status (*P* = 0.82), oestrogen receptor status (*P* = 0.23) or P53 immunoreactivity (*P* = 0.80). The breast-cancer specific 5-year survival for women with I1307 K polymorphism was 88.9% compared with 81.6% in women without I1307K (*P* = 0.34). Using microdissected samples and direct sequencing, no somatic mutations were observed in any of the 24 I1307K-positive cases. Single-strand conformation analysis of 158 of the I1307K-negative breast cancers that were available for study revealed no mobility shifts. We conclude that the presence of the I1307K polymorphism does not appear to be associated with any particular clinicopathological feature of breast cancer and importantly, does not affect the prognosis. © 1999 Cancer Research Campaign


*British Journal of Cancer* (1999) **81**(5), 850–854
© 1999 Cancer Research Campaign
Article no. bjoc.1999.0775

### The effect of the I1307K *APC* polymorphism on the clinicopathological features and natural history of breast cancer

**ZQ Yuan[1,2], LR Bégin[4], N Wong[3,5,7], J-S Brunet[6], M Trifiro[1], PH Gordon[2], L Pinsky[1,5,7] and WD Foulkes[1,5,7]**

Departments of [1]Medicine, [2]Surgery, [3]Oncology, [4]Pathology and [5]Cancer Prevention Research Unit, Sir MB Davis-Jewish General Hospital, 3755 Cote Ste Catherine, Montreal, Quebec, Canada, H3T 1E2; [6]Centre for Research in Women's Health, University of Toronto, Toronto, Ontario, Canada; [7]Departments of Medicine and Human Genetics, McGill University, Montreal, Quebec, Canada

**Summary** The I1307K polymorphism in *APC* has been found to predispose to colorectal cancer in Ashkenazi Jews, and has recently been associated with an increased risk for breast cancer in the same population. In that study, we genotyped 205 paraffin-embedded breast cancers from Ashkenazi Jewish women diagnosed below the age of 65. We now present an extended analysis, with clinicopathological correlations between carriers of I1307K and non-carriers. Twenty-four of 209 cases (11.5%, 95% confidence interval 7.5–16.6) were found to carry the I1307K polymorphism. When stratifying the data by other relevant clinicopathological variables, we observed no association between the presence of this polymorphism and age at diagnosis ($P = 0.52$), grade ($P = 0.074$), tumour size ($P = 0.99$), lymph node status ($P = 0.82$), oestrogen receptor status ($P = 0.23$) or P53 immunoreactivity ($P = 0.80$). The breast-cancer specific 5-year survival for women with I1307 K polymorphism was 88.9% compared with 81.6% in women without I1307K ($P = 0.34$). Using microdissected samples and direct sequencing, no somatic mutations were observed in any of the 24 I1307K-positive cases. Single-strand conformation analysis of 158 of the I1307K-negative breast cancers that were available for study revealed no mobility shifts. We conclude that the presence of the I1307K polymorphism does not appear to be associated with any particular clinicopathological feature of breast cancer and importantly, does not affect the prognosis. © 1999 Cancer Research Campaign

**Keywords:** breast cancer; APC; I1307K; polymorphism; survival

Three founder mutations in *BRCA1* and *BRCA2* account for about 10% of breast cancer occurring in Ashkenazi Jewish women diagnosed at 65 years of age or less (Karp et al, 1997). Other genes, such as *ATM* and *RASH* may play a role in breast cancer susceptibility, but their contribution to breast cancer incidence is presently unknown. The I1307K polymorphism in *APC* has recently been found to be associated with an increased risk of colorectal cancer: the odds ratio (OR) of colorectal cancer in association with this polymorphism was 1.78 for all cases and 2.86 for those diagnosed at under age 66 (Laken et al, 1997). Interestingly, in four of the eight pedigrees illustrated in that paper, possible I1307K-carrying women had been diagnosed with breast (three kindreds) or ovarian cancer (one kindred). We and others have shown that the I1307K polymorphism is over-represented in Ashkenazi Jewish women with breast cancer, compared with ethnically-matched controls (OR = 1.5, $P = 0.003$) (Redston et al, 1998). To further understand the way in which the I1307K allele might increase the risk of breast cancer, we used a historical cohort approach to compare the clinicopathological features of breast cancers occurring in those carrying the I1307K polymorphism in *APC*, with those seen in non-carriers. The effect of the I1307K allele on breast cancer survival was determined.

*Received 3 September 1998*
*Revised 19 April 1999*
*Accepted 13 May 1999*

*Correspondence to:* WD Foulkes

### MATERIALS AND METHODS

### Cases

Study subjects were identified in the medical records department of the Sir Mortimer B Davis Jewish-General Hospital (SMBD-JGH) and included women who self-reported as being Ashkenazi Jewish by birth and who were diagnosed with invasive breast cancer below 65 years of age between 1 January 1986 and 1 November 1995. Two hundred and nine breast cancer blocks from eligible women were identified. All but nine of these samples were used in the primary study (Redston et al, 1998). These samples were rendered anonymous after clinicopathological information was obtained from chart review, and therefore we have no family history available for any of these women. All pathological and molecular analyses of the samples were carried out blinded. The study was approved by the Research Ethics Committee of the SMBD-JGH. Specimens were reviewed by one pathologist (LB). Histological tumour type, grade (1–3) and axillary lymph node status (positive or negative for breast cancer metastases, with the number of positive lymph nodes recorded) was determined by specimen and chart review. The specimens were then coded and DNA was extracted from the paraffin wax-embedded blocks using standard techniques. We used tumour tissue as a source of DNA, and it is possible that I1307K polymorphisms detected could represent somatic mutations. However, loss of heterozygosity (LOH) is not common on 5q in breast cancer (Thompson et al, 1993; Devilee and Cornelisse, 1994; Medeiros et al, 1994) and no shortened forms of the APC protein were observed in cell lines from patients with breast cancer (Smith et al, 1993). Both these observations suggest that somatic *APC* mutations are rare.

### Allele-specific oligonucleotide (ASO) hybridization for I1307K *APC* polymorphism

Genomic DNA from the 210 cases was amplified for polymerase chain reaction (PCR) using primers published previously (Laken et al, 1997). The PCR products were dot-blotted onto nylon filters and hybridized with wild- and mutant type probes at codon 1307. A positive control sample was included on all filters.

### Sequence analysis

Genomic DNA PCR products from samples that were positive by ASO were purified from low-melting agarose gels. Sequences were determined by the dideoxynucleotide-termination method with T7 sequenase Version 2.0 (Amersham Life Science). A positive control was included in each sequencing run. All ASO-positive samples and three ASO-negative samples were sequenced. In all cases, the results were concordant. All 24 positive cases were available for microdissection. Direct sequencing of both malignant and, where possible ($n = 17$) non-malignant regions of the available paraffin-embedded materials was carried out.

### Single-strand conformation analysis

All breast cancer tumours were reviewed by one pathologist (LRB). Specimens where more than 20% of the cells on each section were malignant were chosen for PCR analysis. We chose this figure because dilution experiments demonstrated that we could not detect the I1307K polymorphisms in specimens where less than 20% of the available DNA was derived from a positive control sample. Undissected breast cancer specimens were prepared for PCR. After PCR using published primers (Laken et al, 1997), 1 µl of each reaction mixture was transferred into 6 µl dimethyl sulphoxide (DMSO), loading buffer (30% DMSO, 1 mM EDTA, 0.05% bromophenol blue, 0.05% Xylene cyanol). The samples were heated at 95°C for 5 min and then quickly cooled on ice. Five-microlitres of each mixture was loaded on a 10% non-denaturing polyacrylamide gel (ratio of acrylamide/bisacrylamide 50:1) containing 50 mM Tris–borate (pH 8.3) and 10% glycerol. Electrophoresis was performed using a mini single-strand conformation analysis (SSCA)-gel apparatus at 300 V for 3–4 h at 4°C. After electrophoresis, gels were silver-stained with Silver Stained Plus Kit (Bio-Rad) and dried. The fragment analysed was 115 base pairs long: the A→T polymorphism is present 43 nucleotides downstream from the 3′ end of the upstream primer.

### Oestrogen receptor status

Where available, the conventional radioimmunoassays (RIA) were used to determine oestrogen and progesterone nuclear protein status. These assays were performed at the SMBD-JGH at the time of initial breast cancer surgery using established techniques. A positive oestrogen receptor (ER) or progesterone receptor (PR) score was taken as >10 fmol mg$^{-1}$ protein. In some cases, no record of the RIA could be located, or the diagnostic sample was too small to be studied by RIA, and therefore the ER and PR status was determined by immunohistochemistry using the streptavidin–biotin peroxidase complex methodology. For ER and PR nuclear protein analyses, antibodies 6F11 and 1A6 respectively (Ventana), were used pre-diluted. A positive score was based on >10% cells showing clear, intense nuclear staining, with no background cytoplasmic staining.

### P53 immunohistochemistry

P53 protein accumulation was detected using a standard streptavidin–biotin peroxidase immunohistochemical technique. Anti-P53 (DO-7) monoclonal antibody (Dakopatts) was used at a dilution of 1/50. Four-micron sections were prepared from paraffin-embedded tumour material, and deparaffinized using toluene. Sections were washed in absolute ethanol for 3 min, with two changes, washed in 95% ethanol for 3 min and immersed in tap water for 5 min. Sections were rinsed with distilled water and subsequently immersed in 0.01 M citrate buffer at pH 6.0. Microwave antigen retrieval was performed (5 min × 2) with distilled water immersion. Slides were processed with a Ventana automated system, including the following reagents: hydrogen peroxide (inhibitor), biotinylated Ig, streptavidin HRPO, DAB–hydrogen peroxide copper and haematoxylin. P53 nuclear reactivity was assessed quantitatively as a percentage of nuclei with positive staining among tumour cells and qualitatively in terms of range of staining intensity (+, slight; ++, moderate; or +++, strong). Positivity for P53 accumulation implied 10% of tumour cell nuclei showed immunoreactivity.

### Statistical analysis

#### *Methods*

For the continuous descriptive analysis, we used the *t*-test and the non-parametric Wilcoxon test. For the discrete descriptive analysis, we used Fisher's exact test to calculate the *P*-values and the odds ratio for the corresponding measure of association. The variables were defined as: age (continuous: 28–50, 50–64), tumour size (≤ 2 cm vs > 2 cm), grade (1 vs 2 vs 3), nodal status (0 involved nodes vs 1 or more involved nodes), ER receptor status (negative vs positive) and P53 status (positive vs negative). Confidence intervals were exact binomial probabilities. Kaplan–Meier plots were drawn for breast cancer specific mortality. The log-rank test was used to assess the significance of differences in outcome observed.

#### *Power*

For $\alpha = 0.05$ and $1\text{-}\beta = 0.80$, in this study we could detect ORs greater or equal to 3.3 (tumour size dichotomized), 3.2 (nuclear grade 1 and 2 vs 3), 3.4 (lymph node) and 3.4 (P53) for the association between I1307K positivity and the above-mentioned variables. For the cohort study, we have an 80% power to detect a 29% difference in survival between the carriers and non-carriers of I1307K, with $\alpha$ at 0.05.

### RESULTS

In this series of 209 Montreal Ashkenazi Jewish women with breast cancer diagnosed at less than 65 years of age, we found that 24 women (11.5%, 95% confidence interval (CI) 7.5–16.6) were carriers of the I1307K *APC* polymorphism. No somatic mutations around the I1307K locus were identified by direct mutation analysis of any of the 24 microdissected cases that carried the I1307K polymorphism. In 17/24 cases which were positive for the

© 1999 Cancer Research Campaign

**Table 1** The I1307K polymorphism: comparison with clinicopathological characteristics of cases

| Variable | | I1307K/+ | +/+ (wild-type) | Odds ratio | 95% confidence interval | *P*-value |
|---|---|---|---|---|---|---|
| Age at diagnosis (years) | 28–50 | 8 | 76 | | | |
| | 50–65 | 16 | 109 | 1.39 | 0.57–3.42 | 0.52 |
| Tumour size (cm) | <2 | 14 | 108 | | | |
| | 2–8 | 10 | 77 | 1.00 | 0.42–2.38 | 0.99 |
| Nuclear grade | Low (1) | 2 | 52 | 1.00 | | |
| | Moderate (2) | 13 | 68 | 4.97 | 1.21–20.4 | 0.027 |
| | High (3) | 9 | 65 | 3.60 | 0.81–16.1 | 0.12[a] |
| Axillary lymph nodes[b] | Absent | 15 | 106 | | | |
| | Present | 8 | 67 | 0.84 | 0.34–2.10 | 0.82 |
| Oestrogen receptors[c] | Absent | 4 | 55 | | | |
| | Present | 20 | 129 | 2.13 | 0.71–6.41 | 0.23 |
| p53 immunohistochemistry[d] | Absent | 18 | 136 | | | |
| | Present | 6 | 41 | 1.11 | 0.41–3.0 | 0.80 |

[a]Overall *P*-value: 0.074; [b]12 wild-type and 1 I1307K carriers did not have an axillary dissection; [c]one wild-type case was not studied for ER status; [d]eight wild-type cases were not studied by immunohistochemistry.

I1307K polymorphism, non-malignant tissue was also available. In all cases, the I1307K polymorphism was also present in these cells, thus confirming that the variant observed was present in the germline. Because of data suggesting that the mutated allele might be a site for further somatic mutations, we were concerned that these mutations might have destroyed the ASO-specific sequence, rendering it unavailable for hybridization. However, no alterations were observed in 158 undissected cases that were wild-type by ASO for whom we had remaining tissue available.

We stratified the data by relevant clinicopathological variables such as age at diagnosis, grade, tumour size, axillary nodal status, ER status and P53 immunoreactivity. There was a higher frequency of the I1307K polymorphism in higher grade breast cancers, but the trend was not significant ($P = 0.074$) (Table 1). Because we observed this trend, we stratified the data by *BRCA* mutation status, to determine whether this relationship was confounded by an underlying association between *BRCA* mutation status and grade. In fact, there was no association between grade and I1307K status in *BRCA* mutation carriers (overall $P = 0.62$, data not shown) and in non-*BRCA* carriers, the OR for high grade versus low grade was 1.60 (95% CI 0.26–9.9, $P = 0.68$). The only significant difference in any group was seen for non-*BRCA* mutation carriers, where I1307K carriers were significantly more likely to be grade 2 rather than grade 1 (OR 5.0, 95% CI 1.23–21, $P = 0.04$) but this was only of borderline significance. There was no association between the histological type of the breast cancer and the presence of the I1307K polymorphism, with 75.8% of *APC* wild-type and 70.8% of I1307K carriers having the common ductal type of invasive breast cancer. While it is clear that this study has limited power (~40%) to detect ORs of 2 or less, there is no dramatic association between the I1307K polymorphism and any variable we studied. This is in contrast with the situation for *BRCA1*, where using a subset of the cases reported here, we have shown that *BRCA1*-positive status is highly correlated with high-grade, ER-negative and P53-positive breast cancers (Karp et al, 1997; Foulkes et al, manuscript submitted).

We then studied the effect of the I1307K polymorphism. Not surprisingly, given the absence of any clear association between this polymorphism and clinicopathological variables, breast cancer specific 5-year and 8-year survival did not significantly differ between the I1307K carriers and non-carriers (Figure 1).

### DISCUSSION

A mis-sense mutation in *APC* was identified in a 39-year-old Ashkenazi Jewish man with eight colorectal polyps (Laken et al, 1997). His father had colorectal polyps and his paternal grandmother had both colorectal cancer, diagnosed at age 60 and ovarian cancer at age 72. There was no evidence of FAP. The polymorphism seems to predispose to cancer not as a direct effect on the protein, but by rendering this region of *APC* hypermutable. The polymorphism was found in 47 of 766 (6.1%) Ashkenazi Jewish individuals who were not known to have colorectal cancer, but for whom family history was unavailable. It was also detected in 22 of 211 (10.4%) Ashkenazi Jewish individuals with colorectal cancer for whom family history was available, 172 of which were unselected for family history. Other cancers seen in these families included breast, ovarian, prostate, uterine and throat cancer. Three of the eight illustrated kindreds had at least one case of breast cancer.

In contrast to this positive finding, in 264 individuals from 158 Ashkenazi Jewish breast and breast/ovarian cancer kindreds there was no excess of colorectal cancer in either those who carried the I1307K polymorphism ($n = 12$) or their relatives (Petrukhin et al, 1997). Although the nature of that study precluded the authors from drawing any conclusions regarding I1307K and breast cancer risk in the Ashkenazim, the frequency of the I130K polymorphism was only 4.5% overall (12 positive individuals (11 families) from 264 individuals (158 families)). Therefore it is difficult to argue that the I1307K polymorphism predisposes to breast cancer from their study, as the 4.5% frequency is lower than seen in the controls in the Laken et al (1997) study.

In a large collaborative study an increased prevalence of the I1307K polymorphism in Ashkenazi Jewish women with breast cancer compared with controls was observed (OR = 1.5, 95% CI 1.1–2.0, $P = 0.003$) (Redston et al, 1998). This effect was mainly, if not entirely, limited to women who also carried *BRCA* mutations. In this present study, we analysed the Montreal cases from that publication to determine the relationship between the presence of the I1307K polymorphism and other clinicopathological variables that were available to us (Table 1). The absence of any significant associations, and the fact that we observed no somatic mutations by direct sequencing or by SSCA, suggests that if the

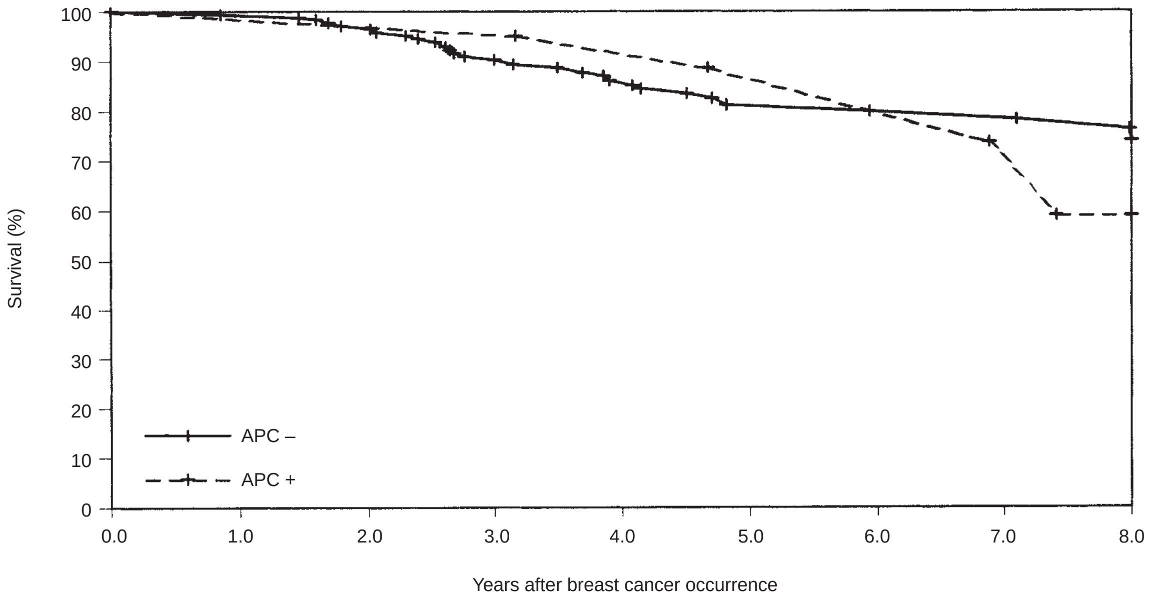

 © 1999 Cancer Research Campaign n at risk

| | | | | | | | | | |
|---|---|---|---|---|---|---|---|---|---|
| APC– : | 185 | 179 | 155 | 122 | 102 | 76 | 58 | 46 | 40 |
| APC+ : | 24 | 23 | 22 | 21 | 18 | 14 | 9 | 5 | 3 |

| | deaths | | % survival | | |
|---|---|---|---|---|---|
| year | APC– | APC+ | APC– | APC+ | P–value |
| 5 | 25 | 2 | 81.6 | 88.9 | 0.3368 |
| 8 | 29 | 4 | 74.4 | 59.3 | 0.8750 |

**Figure 1** Kaplan–Meier survival curves with regard to survival until death from breast cancer are shown for heterozygotes for the 1307K polymorphism (—) and wild-type individuals (– – –). The number of people remaining at risk at the end of each year is shown beneath the figure. The number of events and the survival until death from breast cancer for the two subgroups is shown

I1307K polymorphism does confer an increased risk of breast cancer, it is through a novel mechanism. Genes that confer increased risk of cancer often are associated with a particular phenotype. For example, *BRCA1* mutation carriers develop breast cancers at a younger than average age and the tumours that occur are more likely to be high-grade and ER-negative, even taking into account that they occur in younger women (Karp et al, 1997). Likewise, HNPCC-associated colorectal cancers are more likely to be mucinous and situated in the right colon than their sporadic counterparts (Lynch et al, 1993). The I1307K polymorphism has not been associated with any particular colorectal phenotype, other than a possible earlier age of onset. In Laken et al (1997) there were no grade or stage data provided for the colorectal cancers. Gryfe et al (1999) studied 476 Ashkenazi Jewish patients with colorectal cancer. I1307K carriers had more adenomas and cancers per person than non-carriers, and were diagnosed at a slightly younger age (*P* 0.03). The data in this series of 209 breast cancers indicate that there are no special features of the breast cancers that occur in I1307K carriers.

There have been several 5q LOH studies in breast cancer. The two studies specifically studying markers within, or adjacent to *APC* found modest levels of LOH, in the range 11–28% (Thompson et al, 1993; Medeiros et al, 1994). Allelotype studies including at least one marker on 5q have failed to reveal high levels of LOH (Devilee and Cornelisse, 1994). There are no reported studies of *APC* mutation testing in women with breast cancer, probably because breast cancer is not a feature of familial adenomatous polyposis (FAP). Nevertheless, the low level of LOH on 5q would support the viewpoint that *APC* is not commonly a target for mutations in sporadic breast cancer. The absence of truncated *APC* proteins in seven breast cancer cell lines also indicates that truncating *APC* mutations are unlikely to be common (Smith et al, 1993). However, animal models of FAP have provided another perspective. Min is a mutant allele of the murine *Apc* locus, and is a murine model for human FAP. Interestingly, B6 Min/+ females develop breast tumours, albeit at a low rate (5%), which increases in mice exposed to ethylnitrosurea (Moser et al, 1995). A complicating factor is that the Min allele encodes a stop at codon 850, resulting in a truncated protein, like most *APC* mutations that cause FAP. In contrast, the I1307K polymorphism does not result in a truncated protein. So it is uncertain if there is any biological connection between the breast cancers we observed in I1307K polymorphism carriers in this study and breast tumours in Min mice.

LOH on 5q is common in colorectal cancer, but it is not clear from Laken et al (1997) whether LOH was present in the 23 colorectal cancers from the I1307K-positive individuals that were analysed. In 11, the I1307K allele had suffered somatic mutation centred on codon 1307, but it is not stated whether LOH of the wild-type allele had occurred in these or in the 12 I1307K-positive samples that did not have somatic mutations around this site (Laken et al, 1997). In this study, we sequenced 24 microdissected tumour samples that carried the I1307K polymorphism. The mutations appeared to be germline in origin, with no evidence of somatic instability. In addition, to rule out the possibility that somatic mutations might be present that might destroy the ASO

*© 1999 Cancer Research Campaign* 

site (and thus be recorded as false negative by ASOH), we analysed 158 *APC* wild-type cases by SSCA but no variants were observed (data not shown).

Overall, this study suggests that the I1307K *APC* polymorphism is not associated with any particular clinicopathological features of breast cancer and, in particular, does not affect survival. The significance of this variant for breast cancer risk in the Ashkenazim remains to be fully determined.

### ACKNOWLEDGEMENTS

We thank Prof. B. Vogelstein for providing us with the I1307K-positive control sample, Drs Steven Narod and Michael Pollak for advice, Dr S Karp for his help in initiating this project and Tomasz Stanko and Corinne Serruya for their help in preparing the manuscript. This study was funded in part by a Family Cancer Network Grant from the Fonds de la recherche en Santé du Québec and by the Judy Steinberg Research Fund.

### REFERENCES

Devilee P and Cornelisse CJ (1994) Somatic genetic changes in human breast cancer. *Biochim Biophys Acta* **1198**: 113–130

Gryfe R, Di Nicola N, Gallinger S and Redston M (1998) Somatic instability of the APC I1307K allele in colorectal neoplasia. *Cancer Res* **58**: 4040–4043

Gryfe R, Di Nicola N, Lal G, Gallinger S and Redston M (1999) Inherited colorectal polyposis and cancer risk of the *APC I1307K* Polymorphism. *Am J Hum Genet* **64**: 378–384

Karp S, Tonin PN, Begin LR, Martinez JJ, Zhang JC, Pollak MN and Foulkes WD (1997) Influence of BRCA1 mutations on nuclear grade and estrogen receptor status of breast cancers in Ashkenazi Jewish women. *Cancer* **80**: 435–441

Laken SJ, Petersen GM, Gruber SB, Oddoux C, Ostrer H, Giardiello FM, Hamilton SR, Hampel H, Markowitz A, Klimstra D, Jhanwar S, Winawer S, Offit K, Luce MC, Kinzler KW and Vogelstein B (1997) Familial colorectal cancer in Ashkenazim due to a hypermutable tract in APC. *Nature Genet* **17**: 79–83

Lynch HT, Smyrk TC, Watson P, Lanspa SJ, Lynch JF, Lynch PM, Cavalieri RJ and Boland CR (1993) Genetics, natural history, tumor spectrum, and pathology of hereditary nonpolyposis colorectal cancer: an updated review. *Gastroenterology* **104**: 1535–1549

Medeiros AC, Nagai MA, Neto MM and Brentani RR (1994) Loss of heterozygosity affecting the APC and MCC genetic loci in patients with primary breast carcinomas. *Cancer Epidemiol Biomark Prevent* **3**: 331–333

Moser AR, Luongo C, Gould KA, McNeley MK, Shoemaker AR and Dove WF (1995) ApcMin: a mouse model for intestinal and mammary tumorigenesis. *Eur J Cancer* **31A**: 1061–1064

Petrukhin L, Dangel JE, Vanderveer L, Costalas J, Bellacosa A, Grana G, Daly M and Godwin AK (1997) The I1307K APC mutation does not predispose to colorectal cancer in Jewish Ashkenazi breast and breast-ovarian cancer kindreds. *Cancer Res* **57**: 5480–5484

Redston M, Nathanson KL, Yuan ZQ, Neuhausen SL, Satagopan J, Wong N, Yang D, Nafa D, Abrahamson J and Oczelik H (1998) The APC I1307K allele and breast cancer risk. *Nature Genet* **20**: 13–14

Smith KJ, Johnson KA, Bryan TM, Hill DE, Markowitz S, Willson JK, Paraskeva C, Petersen GM, Hamilton SR, Vogelstein B et al (1993) The APC gene product in normal and tumor cells. *Proc Natl Acad Sci USA* **90**: 2846–2850

Thompson AM, Morris RG, Wallace M, Wyllie AH, Steel CM and Carter DC (1993) Allele loss from 5q21 (APC/MCC) and 18q21 (DCC) and DCC mRNA expression in breast cancer. *Br J Cancer* **68**: 64–68

 *© 1999 Cancer Research Campaign*
